# Editorial: RNA Biology of Microorganisms

**DOI:** 10.3389/fmicb.2021.754109

**Published:** 2021-10-29

**Authors:** Omar Orellana, Orna Amster-Choder, Rajat Banerjee, Jiqiang Ling

**Affiliations:** ^1^Program of Cellular and Molecular Biology, Faculty of Medicine, Institute of Biomedical Sciences, University of Chile, Santiago, Chile; ^2^Department of Microbiology and Molecular Genetics, Faculty of Medicine, The Institute for Medical Research, Israel-Canada (IMRIC), The Hebrew University, Jerusalem, Israel; ^3^Department of Biotechnology and Dr. Bires Chandra Guha Centre for Genetic Engineering and Biotechnology, University of Calcutta, Kolkata, India; ^4^Department of Cell Biology and Molecular Genetics, The University of Maryland, College Park, MD, United States

**Keywords:** mRNA, sRNA, tRNA, canonical and non-canonical roles, degradation, interactions

The intention behind the Research Topic on RNA Biology of Microorganisms was to provide a broad view of the roles of RNA in the physiology of microorganisms, rather than focusing on specific functions. This purpose was mostly accomplished, since the published articles cover subjects from: (i) the life cycle of RNA transcripts, particularly of mRNAs, from birth to degradation; (ii) the various canonical and non-canonical roles of tRNAs, including translation and control of protein fate, as well as influencing genome variability; and (iii) regulatory non-coding RNAs: their identification by novel experimental strategies, the role of redundant RNA versions and the relationship between sRNAs of pathogenic bacteria with host RNAs. Putting all this information together, sheds light on the interplay of events that occur since the birth of an RNA, its distribution in the cell to accomplish its specific roles and its fate upon changes in environmental conditions. In the next paragraphs we briefly describe the contribution of each article to this global view of the biology of microorganisms.

## The Birth, Function, and Fate of Messenger RNAs

Several transcription factors regulate the binding of RNA polymerase to specific transcription promoters, among them NusG, a general transcription factor that is highly conserved in all organisms. Wang and Arstimovitch describe in their review article that NusG plays a number of roles in different cellular contexts, from the classical transcription antitermination of untranslated RNAs by counteracting Rho function on naked RNAs, through stimulation of transcription by avoiding backtracking of RNA polymerase, slowing down of transcription by halting RNAP at certain sequences and connecting RNAP to the ribosome during translation, to recruiting the ribosome to some mRNAs with weak ribosome binding sites. Paralogs of NusG are widely distributed in nature playing roles on specific genes using certain residues at conserved domains (NGN and KOW), while making similar contacts with RNA polymerase through conserved residues.

In a review article presented by Irastortza-Olaziregi and Amster-Choder, a detailed description of the strategies employed by bacterial cells to ensure association between transcription and translation in the apparently not physically separated milieu that encompasses the genetic material and cytoplasm. Physical coupling by direct or indirect interactions (bridged by proteins like NusG) between transcribing RNA polymerase and translating ribosomes are discussed. Lo and behold. Other strategies, such as the control of transcription rate by interaction of (p)ppGpp with RNA polymerase to coordinate transcription and translation, the localization of transcribing DNA to certain locations in the cell away from the bulk nucleoid (at the inner membrane or the surface of the nucleoid) where translation can take place, are also described. However, an important part of the article is devoted to the analysis of emerging data which promote the idea that uncoupled transcription-translation occurs in the context of the apparently non-separated nucleoid-cytoplasm organization of bacterial cells. Several different evidences reveal that this can happen by several tactics, e.g., ribosome free transcription of 5'UTRs, translation-independent localization of mRNAs to different locations in the cell, and faster transcription than translation in *B. subtilis*. A number of strategies, including association of RNA binding proteins (cold shock or ribosomal proteins among other) with the 5′ ends of mRNAs or sRNAs, as well as formation of secondary structures (riboswitches), might aid in the protection of free mRNAs in the cytoplasm against ribonucleases together with delaying translation until an mRNA reaches the proper location. Other proteins that are probably involved in uncoupled transcription-translation are RNA chaperones (Hfq and ProQ).

The review published by Vargas-Blanco and Shell gives a detailed description of the many different mechanisms that give rise to degradation of RNAs, particularly under stress conditions. It is accepted that steady state of RNAs is a balance between synthesis and degradation. Exo- and endo-ribonucleases are responsible for the degradation of RNAs, and their activity can be modulated in several ways to interfere with the RNA substrates, for example, sRNAs and RNA-binding proteins can block the access of RNases. Also, the chemical nature of the 5'end (chemical modifications, such as addition of NAD, Npn, m^6^A or triphosphates) can influence the access of exonucleases. Moreover, secondary structures can also interfere with the access of nucleases. At the 3'end, the formation of poly (A) can facilitate the action of exonucleases. As a general rule, under stress RNAs are stabilized, but their cellular level decreases. This apparent contradiction might be explained by lowering transcription under these conditions.

Setlow and Christie reviewed the data obtained concerning the existence of RNAs in *B. subtilis* spores, as well as other spore-forming bacteria. As opposed to previous assumptions, evidence for the existence of RNAs in spores has been obtained for 1,800 different mRNAs, with no more that 50 mRNAs present in more than 1 copy/spore in the population, with most of them present in <10% of the population. The majority of these mRNAs and rRNAs present in spores are fragmented, and more fragmentation is observed with time post-sporulation. No evidence for the participation of these RNAs in translation after spore germination has been reported, except for *malS* mRNA, coding for malic enzyme, which might provide substrates for the synthesis of ATP. Hence, the data obtained thus far suggest that the main role of RNAs in spores is to provide nucleotides for the synthesis of new transcripts during germination.

## Transfer RNAs in Translation and Evolution

Three articles describe different ways by which transfer RNAs (tRNAs) play unorthodox roles in translation, as well as new roles in processes other than translation. In the article by Arias et al., the authors report that replacement of non-optimal glycine codons by optimal counterparts in the Cdc13 cyclin gene completely impairs the proliferation of cells. Overexpressing the tRNA decoding the non-optimal codons in wild type *cdc13* strains gave rise to severe defects in cell division, accompanied by an increase of Cdc13 aggregation, without affecting the levels of mRNA. The authors propose that the presence of rare glycine codons in *cdc13* plays a role in adjusting the translation efficiency of Cdc13 mRNA to a level competent with proper folding of the protein to guarantee its functionality.

A review article by Krahn et al. points to non-canonical structures formed by tRNAs. The classical and well-known secondary structure of tRNAs resembles a clover leaf, in which the acceptor stem, D loop arm, anticodon arm, variable arm and TφC arm are conserved between all organisms. However, some variations of this conserved structure have been shown to accomplish specific roles in the charging and translation of specific amino acids that are decoded by specialized termination codons. Examples, provided by selenocysteinyl, pyrrolysyl-tRNA, and others, show that these tRNAs are aminoacylated by canonical amino acids, which are later transformed into the derivatives that are incorporated to proteins. These tRNAs show extended acceptor, TφC and D stems. These modification are relevant for their recognition by the enzymes that modify the amino acids, as well as by the specialized translation elongation factors that allow recognition of the specific coding termination codons, thus facilitating the incorporation of these amino acids into proteins. Special emphasis is placed on the deviation of mitochondrial tRNAs from canonical structures, where different versions are found, including the lack of either the TφC or the D arms, or both, in tRNAs that are still functional in nematodes.

Other dimensions of tRNAs utility are highlighted in the review article by Guimarães et al. They depict the other roles, beside translation, of tRNA and their genes (tDNAs). The authors first describe the participation of transcription factor TFIIIC, one of the RNA polymerase III general transcription factors, which transcribes tDNAs (and 5S rRNA) in eukaryotes by recognizing internal transcription promoters. The transcription machinery helps in the recruitment of architectural chromatin proteins, such as condensin and adhesin, that help limiting the spreading of heterochromatin into the active euchromatin. A second case described by the authors is the effect of tRNAs on genome stability. The tDNAs are known as sites where R loops are formed, particularly when replication collides with transcription of tDNAs. These R loops are precursors of genome instability. Additionally, tDNAs in yeast, as well as in bacteria, are prone to insertion of transposable elements (retrotransposons). Besides the insertion of these genetic elements, they create a framework for duplications or deletions of segments of the genome within inserted genetic elements. Another interesting effect of tRNAs on genome stability and evolution occurs in yeast when tRNA pools are altered, generating variability in the decoding capacity of certain tRNAs, imposing consequences on proteome stability. In the long run, these effects cause instability of chromosomes, particularly in regions encoding stress response genes as adaptive evolutionary events.

## Non-Coding RNAs as Regulatory Elements

During the last couple of decades, a vast number of publications described the identification of non-coding RNAs as molecules that regulate gene expression. In this Research Topic, four articles, all reporting original research, expose different aspects of the regulatory role of non-coding RNAs. The articles by Li et al., and by El Mouali et al. describe studies of duplication of regulatory RNAs and their roles in different states of the bacterial life. The first example is that of the two copies of 6S RNAs (6S-1 and 6S-2) from *Bacillus thuringensis* that are co-transcribed from an operon, whose transcript requires processing. The deletion of the gene encoding 6S-1, and not 6S-2, affects bacterial growth in stationary phase, as well as spores production. The role of the 6S-2 is not known yet. The second example is that of the two copies of the RNAs, CsrB, and CsrC, that regulate the function of CsrA, the protein controlling translation of mRNAs of the carbon storage regulon. These regulatory RNAs, existing in a number of bacteria, bind to CsrA, titrating the protein and thus avoiding its interaction with GGA motifs in the target mRNAs. While CsrB is constitutively expressed in *Salmonella*, CsrC is regulated at the transcription level by the master carbon regulator CRP-cAMP. It is repressed at the logarithmic phase with the contribution of Spot42, an sRNA that is also controlled at the transcription level by CRP-cAMP.

Novel technologies for global analysis of RNAs have uncovered a vast variety of small non-coding RNAs (sRNAs) that play diverse functions in the regulation gene expression, mostly at the post-transcriptional level by binding to target mRNAs and altering their fate, both in bacteria and in eukaryotes. First identified as curiosities decades ago and later, due to advancements in bioinformatics and genome sequencing, many RNAs were predicted and identified in intergenic regions. Recent development of RNA-seq-accompanied methodologies have made possible the identification of many more sRNAs, in both intergenic and within coding regions of various organisms. Different approaches for isolating and identifying sRNAs in non-coding and coding regions of genomes have been developed. Their interaction with certain proteins has made it feasible to isolate and identify bound sRNAs and their regulated target mRNAs, permitting the prediction of their function in the cell. The article by Bar et al. describes a new computational approach, which uses machine-learning-based algorithm for the prediction of novel sRNAs, to define previously unknown sRNAs found by the recently developed RIL-sec technology for identifying sRNA that bind to the Hfq chaperone. Currently, around 2,800 sRNAs in *E. coli* have been found to interact with Hfq. The ~1,000 identified in this work are encoded by different regions in the genome, either from intergenic regions or as part of the sense or antisense coding sequences in mRNAs transcripts.

Interest in the interaction between commensal bacteria and their host(s) has increased in recent years, mainly due to the analysis of the human microbiome. The article by Legüe et al. sheds light on the interaction between the model worm *C. elegans* and bacterial pathogens. The authors report that chronic exposure to the pathogen induces diapause formation (PIDF) in *C. elegans*. This defense mechanisms is transgenerational, that is, it is inherited by the worm and can be recalled upon exposure to pathogens a few generations forward. This mechanism requires the RNA interference machinery of the worm and sRNAs from the pathogenic bacteria. The authors used bioinformatic analyses of transcriptomic data from holobionts (bacteria and worm) to predict intergenerational and transgenerational interactions of sRNAs from both organisms. Their data points at pairing of RNAs from the two organisms, RNAs with similar sequences from the two organisms and eukaryotic sequence motifs found in bacterial RNAs. Surprisingly, chemical modification of tRNAs emerged as crucial events controlling the formation of the defensive form of the worm and, hence, relevant for the PIDF.

## Concluding Remarks

The topics described in this Research Topic expose the many faces, roles and occurrence of RNA molecules. These studies show that the dynamic changes in mRNAs level toward a steady state concentration, which allow the cell to balance gene expression, is accomplished at the level of transcription, finely tuning the activity of RNA polymerases, but also by controlling the decay of RNAs. Also shown is the interplay between mRNAs and ribosome in bacteria, which is not limited to coupled transcription-translation, but is also relevant for uncoupled processes that allow adaptation to specific cellular requirements. Transfer RNAs, for a long time regarded as vehicles for transporting amino acids to the ribosome during translation, are emerging as active regulators of gene expression, making codon usage a tool for responding to specific physiological events, as well as molecules that play roles in evolutionary processes at the level of genome stability. The diversity of small non-coding RNAs continues to unveil, and their ability to play important regulatory roles is found not to be constrained to particular organisms, but to extend to the interaction of bacteria with their eukaryotic hosts. Together, these articles bring many exciting findings that sheds new light on bacterial RNA biology.

## Author Contributions

All authors listed have made a substantial, direct and intellectual contribution to the work, and approved it for publication.

## Funding

This work was supported by Grants No. 1274/19 from the Israel Science Foundation (ISF) founded by the Israel Academy of Sciences and Humanities to OA-C, DST-SERB (order no. EMR/2016/002247), Govt of India to RB, National Institute of General Medical Sciences R35GM136213 to JL, and Fondecyt, Chile 1190552 to OO.

## Conflict of Interest

The authors declare that the research was conducted in the absence of any commercial or financial relationships that could be construed as a potential conflict of interest.

## Publisher's Note

All claims expressed in this article are solely those of the authors and do not necessarily represent those of their affiliated organizations, or those of the publisher, the editors and the reviewers. Any product that may be evaluated in this article, or claim that may be made by its manufacturer, is not guaranteed or endorsed by the publisher.

